# Healthy buildings for a healthy city: Is the public health evidence base informing current building policies?

**DOI:** 10.1016/j.scitotenv.2020.137146

**Published:** 2020-06-01

**Authors:** Laurence Carmichael, Emily Prestwood, Rachael Marsh, Janet Ige, Ben Williams, Paul Pilkington, Eleanor Eaton, Aleksandra Michalec

**Affiliations:** a WHO Collaborating Centre for Healthy Urban Environments, UWE Bristol, Coldharbour Ln, Stoke Gifford, Bristol BS16 1QY, United Kingdom of Great Britain and Northern Ireland; b Birmingham Energy Institute at University of Birmingham, Edgbaston, Birmingham B15 2TT, United Kingdom of Great Britain and Northern Ireland; c Department of Health and Social Sciences, UWE Bristol, Coldharbour Ln, Stoke Gifford, Bristol BS16 1QY, United Kingdom of Great Britain and Northern Ireland; d Air Quality Management Resource Centre, UWE Bristol, Coldharbour Ln, Stoke Gifford, Bristol BS16 1QY, United Kingdom of Great Britain and Northern Ireland; e Bath University, Claverton Down, Bath BA2 7AY, United Kingdom of Great Britain and Northern Ireland

**Keywords:** BRE, Building Research Establishment, BREAAM, Building Research Establishment Environmental Assessment Method, BSI, British Standards Institution, BTO, Building Technologies Office, CISBE, Chartered Institution of Building Services Engineers, CSH, Code for Sustainable Homes, DCLG, Department for Communities and Local Government, DEFRA, Department for Food and Rural Affairs, DoH, Department of Health, EHS, English Housing Survey, HES, Mortality and Hospital Episode Statistics, HHSRS, Healthy Housing Safety Rating System, HMSO, Her Majesty's Stationery Office, HSE, Health and Safety Executive, MHCLG, Ministry of Housing Communities and Local Government, NPPF, National Planning Policy Framework, NPPG, National Planning Policy Guidance, ODPM, Office of the Deputy Prime Minister, PHE, Public Health England, SDGs, Sustainable Development Goals, Housing, Health, Hazards, Evidence base, English planning, Building regulations

## Abstract

Research has demonstrated that housing quality is a key urban intervention in reducing health risks and improving climate resilience, addressing a key ambition of the United Nations Sustainable Development Goals. Yet housing quality remains a problem even in high income countries such as England. In particular, hazards such as excess cold, excess heat and lack of ventilation leading to damp and mould have been identified as a major issue in homes. Research shows that these hazards can lead to a range of health conditions, such as respiratory and cardiovascular disease, infections and mental health problems. This article explores the use of public health research and evidence in policy to regulate new buildings in England to deliver improved public health, climate resilience and a reduced carbon footprint, in particular exploring the policy drivers and awareness of the public health evidence.

Findings show that public health evidence is hardly referenced in policy and that the focus on other evidence bases such as on climate mitigation in building regulations results in both positive and negative impacts on health. This reflects a lack of a systems approach around urban interventions leading to weaknesses in standards regulating the private development sector. In conclusion, this paper recommends: 1. the consideration of health impact in future building regulations; 2. the integration and coordination of key policies covering various scales and phases of the development processes and 3. the better education of residents to understand advances in new energy performance technologies.

## Highlights

• Review of English building policies and regulations revealed gaps in evidence use.
• Building policy in England focuses on climate mitigation rather than public health.
• Research found that building policy uses public health evidence in a patchy way.
• Lack of systems thinking has led to building standards ignoring health.
• A single policy regime must regulate different phases and scales of urban development.

## Graphical abstract

**Figure:**
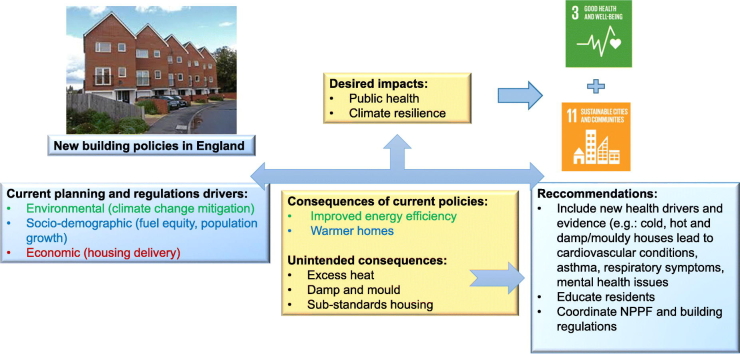

## 1 Introduction

Research for this article was funded by the Wellcome Trust's Sustaining Health programme. The project entitled “Upstream” sought to develop new approaches for integrating long-term health outcomes into urban development planning and delivery by using England as a case study (Upstream, 2018).

The United Nations' Sustainable Development Goals (SDGs) offer an overarching framework for improving the environment and health in cities (UN, 2015). Several SDGs and the resulting targets have a built environment dimension, aimed at improving both environmental quality and public health through interventions to enhance urban infrastructure or the quality of housing (Box 1). In particular, the SDGs indicator 11.1.1 explicitly refers to the need for adequate housing standards. Moreover, a number of SGDs are yet to establish implementation methodology and data sources, providing an opportunity for a timely intervention.

Box 1 **Sustainable Development Goals (SDGs) and indicators related to the built environment.**

**Table:** 

Indicator 3.9.1: Mortality rate attributed to household and ambient air pollution Department for Environment, Food and Rural Affairs - DEFRA, 2013 UK data context: Percentage of adult deaths (aged 30 and over) attributable to particulate air pollution dataset from the Department for Environment, Food and Rural Affairs (DEFRA) (English dataset only) Connection to built environment: Although the dataset groups household and ambient air pollution in one indicator, well-evidenced connections between housing quality and pollution will affect the performance of this measure. Indicator 11.1.1: “Proportion of urban population living in slums, informal settlements or inadequate housing” UK data context: Percentage of dwellings failing minimum standard decent homes criteria (English dataset only) Connection to built environment: This indicator looks directly into the quality of housing based on the UK policy tool Housing Health and Safety Rating System (HHSRS) Indicator 11.a.1: Proportion of population living in cities that implement urban and regional development plans integrating population projections and resource needs, by size of city UK data context: methodology not yet established Connection to built environment: This indicator pertains to the quality of strategic planning at the urban or regional level and therefore could inform the quality and quantity of housing built in the future. As the implementation methodology has not been established, there is an opportunity for the researchers to advise on the choice of data. Source: ONS, 2019

Alt-text: Box 1

SDGs respond to the mounting evidence highlighting the links between our health and the living environment. In particular, a strong body of international public health literature is giving a fuller understanding of the impact design features at the building scale can have on health and identifies a range of health hazards in the home Research in high income countries has identified 14 actionable urban planning principles associated with improved health and wellbeing including enhanced neighbourhood walkability, increased provision of affordable and diverse housing and improved quality of housing (Bird et al., 2017). Further research in the Upstream project identified important associations between thermal quality, ventilation, housing affordability, safety and wellbeing of residents (Ige et al., 2018). These findings show that sub-standard housing is not the monopoly of low- and medium-income countries. In England, policy-makers are all too aware of this. The damning conclusion of the independent Hackitt Review, for instance, declared that building regulations are ‘unfit for purpose’. Although the report relates more specifically to fire safety following the Grenfell Tower tragedy in London, the statement highlights the urgent need for regulation and policy to better recognise the interdependencies between different parts and ensure that buildings are both fit to tackle climate change and to support human health (MHCLG, 2018a).

Statistics further support that housing significantly affects human health in England. In 2017, 4.5 million homes (19%) in England did not meet the Decent Homes Standard, a policy tool used to assess the condition of the existing UK building stock through the yearly English Housing Survey (EHS). The survey accounts for a variety of criteria, including the provision of a reasonable degree of thermal comfort (Department for Communities and Local Government (DCLG), 2006a, Department for Communities and Local Government (DCLG), 2006b). In addition, 11% of English homes are experiencing “*serious and immediate risk to a person*'*s health and safety*” (MHCLG, 2019).

The high number of below standard homes is likely to have substantial associated health costs. The Building Research Establishment (BRE) calculated that the cost to the NHS alone is some £1.4billion per year to treat people living in the poorest^1^ housing in England (BRE, 2015). Drawing from the outcomes identified in the systematic evidence review identifying strong evidence of impact of health hazards at building level (Ige et al., 2018), researchers have calculated that in the UK: 1. the total average annual cost of cold, excess heat and damp and mould per population of 1000 people could be respectively £240,500, £470,000 and £325,000 (Upstream, 2018). These figures remain tentative and reflect uncertainties in calculating impact on the risk of illness or odds ratios observed in the medical evidence, and uncertainties in the valuations related to the severity and duration of illness (Upstream, 2018). However, to place these figures into context, OECD estimated that in 2016 total healthcare expenditure in the UK per capita was £2892 (OECD, 2019 converted from USD).

This article aims to analyse the pathways between evidence and housing regulation and policy in England (Including the EU legislation applying to England at the time of writing) with three housing health hazards (damp and mould, excess cold and overheating) set out in the HHSRS (DCLG, 2006b). The reason for choosing hazards from the HHSRS list of hazards is developed in the theory section. In particular, the research team was interested in exploring the mismatch between the evidence currently available on these three hazards and the evidence used to inform policy around buildings.

## 2 Hypothesis and approach

### 2.1 Sub-standard housing in England: market failure

The existence of sub-standard housing in England can be seen as a market failure to deliver required standards. Market failure leads to an inefficient allocation of resources and is demonstrated in England through homelessness (even though there are empty homes), the high cost of housing, the number of affordable homes included in new developments consistently falling below local authority targets and design quality issues even in new homes. Key to this market failure even in new developments is the legitimate use of viability assessment findings by developers to reduce the number of affordable homes, quality of the design, or size of the homes they are required to build. If profits are predicted to fall below 20% then developers can reduce their commitments in negotiations with local authorities at the planning stage to ensure the future commercial viability of the development.

### 2.2 Recognising public health evidence in housing policy

This article argues that the market failure to deliver homes in sufficient numbers, quality and affordability is underpinned by the failure to comprehensively reflect the public health evidence in policy and regulation. Because health evidence is not systematically acted upon in policy, the health impact of sub-standard homes is not sufficiently recognised in negotiations between developers and local authorities. In addition, this article argues that the current lack of a systemic approach around urban health interventions is leading to weaknesses in standards regulating the private development sector. In particular, this article focuses on the contrasting positive and negative health impacts from improvements in design and build quality of new homes to reduce energy consumption and tackle fuel poverty. Thermal comfort is improved while at the same time problems with damp, mould, overheating and adequate ventilation are exacerbated due to increased insulation and air-tightness levels. A systematic approach is required that reflects the need to consider both energy efficiency and health. A review of building regulations across Europe found that every country studied had similar structures for building control systems and technical requirements (Branco Pedro et al., 2010), meaning these findings are likely to be transferable to other countries.

### 2.3 Damp and mould, excess cold, and overheating as key hazards for health in homes

The research presented in this article centres on three health hazards in the Healthy Housing Safety Rating System (HHSRS) associated with poor building design and the thermal performance and quality of housing: damp and mould, excess cold, and overheating. The 2017–18 English Housing Survey shows that 4% of English homes had damp, 2% had problems with condensation and mould and 7% of residents also reported their homes as uncomfortably hot (MHCLG, 2019). In Europe this problem is even greater, with the EU statistics on income and living conditions from 2016 show that 15.4% of homes had damp and 8.7% of homes were not able to stay adequately warm (Eurostat, 2018). We saw above that research is starting to put a health cost on these hazards (BRE, 2015; Upstream, 2018).

The policy analysis focuses on these health hazards in particular due to the size and quality of the existing evidence base identified in the project's systematic review linking the thermal quality of buildings with health and well-being in high income countries (Ige et al., 2018). Though thermal quality issues such as mould and damp are often associated with older homes, thermal quality of housing is an issue in *new* buildings in England as mentioned above. Arguably, regulations on new and existing buildings have been less successful in supporting broader health outcomes, despite building energy performance standards having improved over the years in response to climate change mitigation and fuel poverty rising up the policy agenda.

A number of studies conducted outside the UK have called for better consideration of thermal quality in building design and regulations (Howden-Chapman et al., 2008; Healy, 2003). Findings from Healy, 2003 showed that four European countries with the poorest standard of housing, Portugal, Greece, Ireland, and the UK, recorded higher scores for excess winter deaths. A randomised controlled study conducted in New Zealand examined the impact of improved home heating on asthma and respiratory outcomes among children (Howden-Chapman et al., 2008). Findings from this study showed that children living in homes with improved heating had fewer reports of poor health, reported fewer visits to the doctor or pharmacy for asthma related conditions, and had fewer days off school than children who did not receive improvements to the heating system until the end of the trial.

### 2.4 The rational for a more integrated approach to protecting both the environment and human health

Policy had already identified them as risk factors (HM Government - Housing Act, 2004; Department for Communities and Local Government (DCLG), 2006a, Department for Communities and Local Government (DCLG), 2006b). But a lack of integrated thinking in regulating new building quality has led to an uneven system favouring climate change mitigation at the expense of adaptation and securing broader health outcomes. As previous research shows, buildings in developed countries are becoming increasingly airtight as a response to stricter energy efficiency requirements (Milner et al., 2011; The findings section will retrace the evolution of policy drivers of building policy). This article therefore argues for a more systems-based approach to building policy that would consider both human health and climate change. Systems approaches are increasingly used to explain the interconnections between the built environment and health. The socio-environmental approach to health developed by Dahlgren and Whitehead (Dahlgren and Whitehead, 1991) has identified a complex web of social, economic and environmental risk factors on health and health equity. In particular, the link between our health and living environment has been well documented over the years at all scales from homes to city scale (Barton and Grant, 2006; Carmichael et al., 2019; Barton, 2017; Barton et al., 2015; Corburn, 2013). The socio-environmental approach has been developed further to link ecosystem conditions (biophysical, chemical or biodiversity) to wellbeing (Reis et al., 2015). This latest model is useful to advocate interventions at international/national (e.g. eliminating diesel engines) and local (e.g. urban planning) levels, it also advocates for the considerations of the co-benefits of an intervention (e.g. energy efficiency AND wellbeing). It is, however, not necessarily easy to translate the model to the real world. A number of issues emerge: 1. the strength of the evidence varies for different risk factors (e.g. PHE, 2017; Ige et al., 2018); 2. apportioning health risks to various factors is a complex issue within a system (e.g. Government Office for Science, 2007) and 3. regulatory regimes remain siloed with different scales of the built environment ruled by different sets of standards and regulations, reflecting different disciplines, professions, practices and policies (Siri et al., 2016). Yet, these can have a combined effect as they are linked (e.g. instance urban planning delivering homes and promoting sustainability but not regulating building fabric (Ige et al., 2018)) and research and practice should seek solutions which support human health as well as the environment.

## 3 Materials and methods

### 3.1 Identifying the current evidence base on the impact of buildings on health

**Fig. 1:**
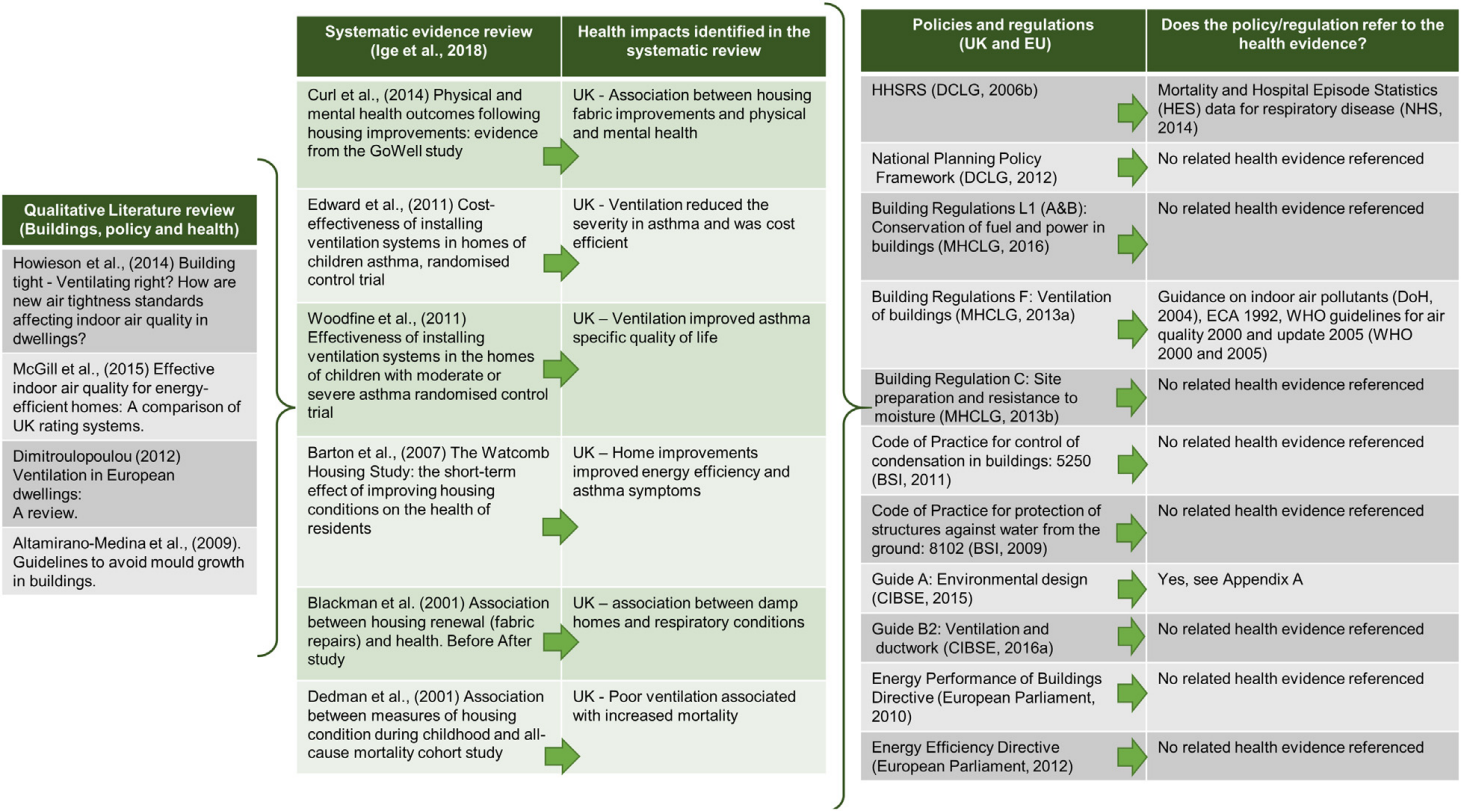
Evidence pathway for housing hazard ‘damp and mould growth’.

**Fig. 2:**
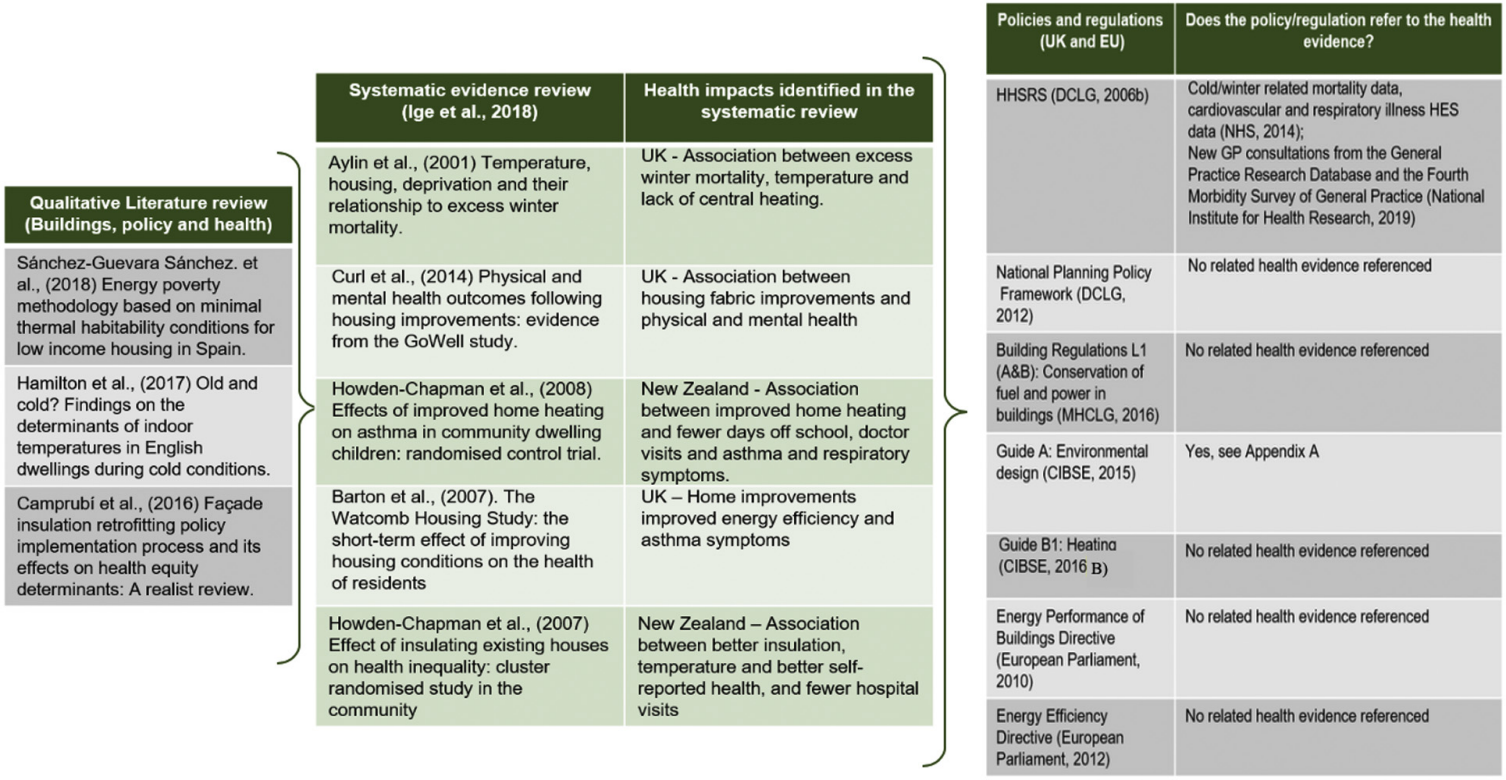
Evidence pathway for housing hazard ‘excess cold’.

**Fig. 3:**
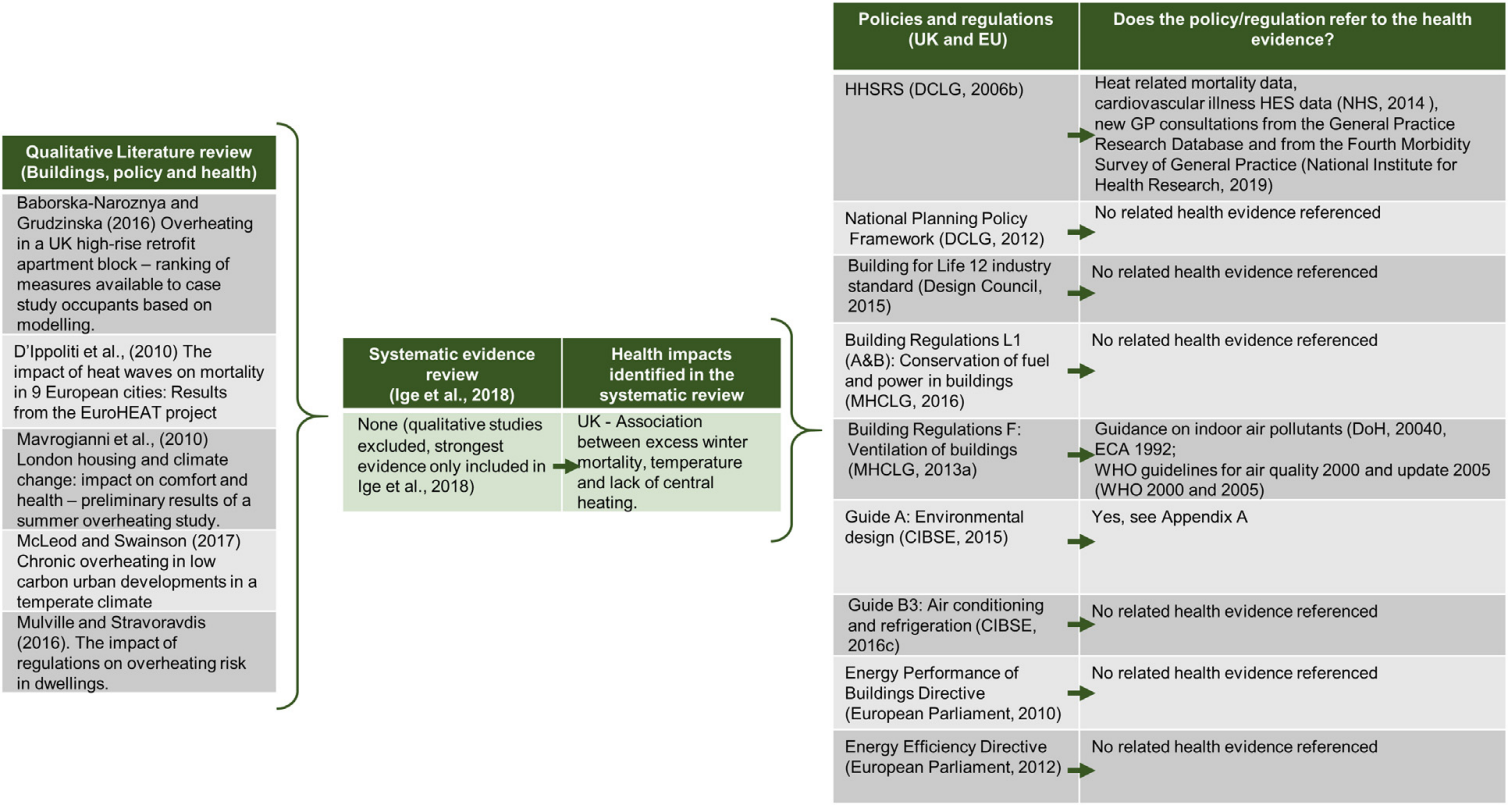
Evidence pathway for housing hazard ‘excess heat’.

The Upstream project provided the literature review on the impact of design features on health at the building scale. This article used only the evidence associating buildings' thermal quality and health that was identified in this review and listed in Fig. 1, Fig. 2, Fig. 3^2^ (​Aylin et al., 2001​; Baborska-Naroznya and Grudzinska, 2016; Barton et al., 2007; Curl et al., 2014; Department of Health, 2004; Dimitroulopoulou, 2012; European Concerted Action, 1992; European parliament, 2010; European Parliament, 2012; Hamilton et al., 2017; Howden-Chapman et al., 2014; McGill et al., 2015; Ministry of Housing Communities and Local Government (MHCLG), 2016; National Health Service, 2014; National Institute for Health Research, 2019; Sánchez et al., 2018; World Health Organisation, 2005; Ige et al., 2018). The full methodology for this evidence review can be found in Ige et al. (2018). Drawing from Ige et al. (2018), the authors undertook qualitative policy review and semi-structured expert interviews. This allowed to contextualise evidence-policy gap and provides a rich description of the current state of knowledge and practice in UK housing.

### 3.2 Qualitative review: identifying policy drivers of key regulations around buildings

The research then wanted to explore if English policy on building was informed by the public health evidence base. First the researchers identified key guidance and regulations on buildings. The initial list of key policies and building regulations (Fig. 1, Fig. 2, Fig. 3) for new housing development in England was elaborated through a full search of relevant governmental department policy libraries, in particular the Ministry of Housing, Communities & Local Government, and identified standards such as CIBSE. Initially, policy documents were reviewed using key-word searches for “health”, “cold” “heat” “damp” “mould”. Policy documents with positive matches to key words were subject to a more in-depth review to identify specific reference to the health evidence base, with in-policy references recorded in a database. Subsequently, in-policy health references were crosschecked with the evidence base identified in the systematic review (Ige et al., 2018) and studies identified in a ‘buildings, policy and health impacts literature review.

Referenced studies in policy documents relating to workplaces and healthcare premises were not included as the emphasis of the analysis was on residential buildings. References related to increased productivity and comfort were also not included as they are not specifically health outcomes.

Once the policies were identified, researchers carried out qualitative review of literature on buildings, policy and health aimed to explore the evolution of drivers influencing English building policy and regulations and the extent to which public health evidence competes with other priorities.

The methodology for the qualitative literature review is as follows. In Scopus, searches were run using the terms: building *and* health *and* (policy *or* regulation *or* standard *or* directive) *and*… with the final term being each of the items in the HHSRS hazard list (Department for Communities and Local Government (DCLG), 2006a, Department for Communities and Local Government (DCLG), 2006b). For each hazard, the results were exported to a separate spreadsheet and labelled before combining, sorting alphabetically and removing duplications. For the analysis in this article, results relating to damp and mould, cold and overheating were exported and screened. Abstracts were screened for relevance and categorised against the following criteria:

• Findings discuss i. the link between a health impact (e.g. asthma, obesity, injury, cancer, heart disease), ii. a building design feature (e.g. ventilation, thermal properties) and iii. a policy/ regulation/ standard/ directive;
• Has a UK or EU focus

Studies were categorised as 1, 2 or 3, with 1 fully meeting both criteria, 2 meeting the first criteria but not being UK or EU focussed and 3 not fully meeting either criteria. Studies categorised as 1 were subject to a more in-depth review of their findings with a focus on articles published since 2000. The choice of this date assumed an emergence of a body of literature on building thermal performance, policy and regulation linked to the adoption of the EU Directive on the Energy Performance of Buildings in 2002.

### 3.3 Semi-structured interviews with stakeholders

Two rounds of 14 semi-structured interviews with practitioners were carried out, confirming the key drivers for building policies and practice (Upstream, 2018). The urban development process is complex, involving a series of different actors with different agendas and no common understanding of the built environment as a determinant of health. The interviewees represented a wide range of key decision-makers from English principal urban development delivery agencies. The interviews with senior executives from the public and private sector sought to explore in particular the practitioners' understanding of health, the importance of health evidence in their decision-making process, barriers and opportunities to the creation of healthy and sustainable urban environments, and agencies and networks for delivering healthy urban sustainable environment. Each interview was undertaken using a framework of 13 thematic areas developed by the research team with input from four expert advisors representing real estate, city government, estate agency and volume house-building. Coding of qualitative interview transcripts used the NVivo software. Interviews helped clarify the role of evidence in the practice of housing delivery and the issues raised by practice around the research/practice synergy (Upstream, 2018).

## 4 Findings

### 4.1 Scope and limit of current policy to regulate damp and mould, excess cold or overheating in new buildings in England

The statutory HHSRS was introduced in 2006 following the Housing Act (HM Government - Housing Act, 2004; Department for Communities and Local Government (DCLG), 2006a, Department for Communities and Local Government (DCLG), 2006b). It changed the way housing conditions were assessed to place the onus on local authorities and to look at the condition of properties using a risk assessment approach rather than a set of minimum standards. It is concerned with avoiding or, at the very least, minimising potential health hazards of which it lists 29. Hazards are classed as category 1 or 2 depending on the likely impact. The HHSRS is supported by extensive reviews of the literature and by detailed analyses of statistical data on the impact of housing conditions on health. It is a system applied to the *existing* housing stock only but can be used to assess housing of any tenure. In practice, it is often used as a reactive safeguarding method, largely adopted for housing in the socially or privately rented sector and often relying on complaints from tenants (House of Commons, 2018). If HHSRS highlights a hazard, a range of policy tools are available for local authorities to use such as providing advice; signposting to other agencies, financial assistance, and only after all informal avenues have been exhausted enforcement action (Planning Portal/MHCLG, 2019a; e.g. South Gloucestershire Council, 2018).

Table 1a, Table 1b, Table 1c summarises the hazards, the associated possible health effects, pathway/cause and housing design defects identified in HHSRS with their data sources.

**Table 1a:** Summary of pathways between damp and mould hazard from the HHSRS and health outcomes.

Hazard	Possible health effect	Pathway/cause	Design feature/defect	Value
Damp and mould	*Asthma *Depression, anxiety, social isolation *Allergy: rhinitis, conjunctivitis, eczema, cough and wheeze *Fungal infection Suppressed immune system	*Reduced ventilation levels *Increased humidity, especially beyond 70% *Warmer indoor temperatures in winter	*Lack of damp proof courses *External fabric allowing rain penetration *Lack of frost protection *Poor bath and sink design *Poorly installed drainage *Poorly installed rainwater goods *Poorly ventilated roof and under floor spaces *Inadequate means of ventilation *Poor extraction of moisture laden air	The health impact potential of damp and mould on respiratory illnesses, eczema and headaches could be valued at £325,000 per 1000 people per year (Upstream, 2018)

**Table 1b:** Summary of pathways between excess cold hazards from the HHSRS and health outcomes.

Hazard	Possible health effect	Pathway/cause	Design feature/defect	Value
Excess cold	Below 19 °C: small risk, Below 16 °C: serious health risks for the elderly, Below 10 °C: great risk *Cardiovascular conditions: stroke, heart disease, hypertension *Respiratory disease *Suppressed immune system	*Changes in outdoor temperature *Low energy efficiency ratings (poor insulation) *Absence of central heating/poor inefficient heating systems *Excessive damp which reduces thermal insulation	*Thermal insulation *Appropriate/properly installed or maintained occupant controllable heating system *Appropriate/properly installed or maintained occupant controllable low-level background ventilation *Means for rapid ventilation at times of high moisture production in kitchens/bathrooms *Properly sited/sized permanent openings (e.g. air bricks/open-able windows) *Properly fitting butt-jointed floor boarding/doors/windows	The health impact potential of cold on mortality, sickness absence, and hospital admissions could be valued at £240,500 per 1000 people per year (Upstream, 2018)

**Table 1c:** Summary of pathways between excess heat hazard from the HHSRS and health outcomes.

Hazard	Possible health effect	Pathway/cause	Design feature/defect	Value
Excess heat	*Thermal stress *Cardiovascular conditions: stroke *Mortality increases in temperatures over 25 °C	*Poor ventilation *Smaller dwellings *Large areas of south facing glazing *Faulty or sub-standard heating controls	*Shuttering or blinds *Natural ventilation or air conditioning *Controllable heating systems	The health impact potential of excess heat on mortality could be valued at £470,000 per 1000 people per year (Upstream, 2018)

Despite this existing policy framework, the thermal quality of new buildings remains an issue. Hence two questions emerge for the future: is the public health evidence comprehensively reflected in policy regulating new buildings and why have some advances been made on health (tackling fuel poverty) while at the same time damp and mould and overheating are emerging more strongly in English housing? Is a lack of a systems approach in building regulations leading to the creation of new health issues?

### 4.2 Thermal quality of buildings on health: the evidence base

Extensive reviews of the literature and statistical data on the impact of housing conditions on health informed the HHSRS which aimed to inform practice at the time of its development (Department for Communities and Local Government (DCLG), 2006a, Department for Communities and Local Government (DCLG), 2006b). However, the guidance recognises the continuing process of the knowledge creation and that “*it is the responsibility of professionals using the HHSRS to keep up*-*to*-*date on current evidence*” (ODPM, 2004, p.7). The Upstream evidence review (Ige et al., 2018) identified 40 studies under the theme of ‘buildings’. Within these, eight studies provided strong to moderate evidence of the impact of design features related to “improved quality of housing (thermal and ventilation)” on health (Fig. 1, Fig. 2, Fig. 3). In particular, the links were strong between building fabrics and excess cold as well as with damp and mould. Weak evidence was identified for excess heat. The HHSRS had similarly identified weak evidence for excess heat. Here, qualitative literature discussing the link between health impacts, building design feature and policy instruments further identified qualitative evidence on the impact of design features linked to thermal comfort and ventilation on health (Fig. 1, Fig. 2, Fig. 3).

### 4.3 Translating the evidence base to the practice of development

When interviewees reflected on the meaning of health for the built environment, they included the thermal quality of homes, in particular damp and mould and the need to ventilate. One developer for instance stated:

“*Having enough houses so that people aren*'*t homeless*, *having an address so that you can then apply for a GP*, *be part of the social fabric of which health is part of collective provision*, *the right not to live in a damp house* (…)”.

Another (public sector developer) mentioned the need for properties to be

“[properties need to be] *cost effective to heat*, *to ventilate*. *Those things are really important*, *so that people can live in a comfortable environment*. (…) *As a council*, (…) *we*'*re trying to reduce the energy costs of the properties*. *Within that*, *within doing passive house*, *you also have to make sure the ventilation*'*s right*. *Because otherwise you can get quite stifling environments*”.

The HHSRS requires professionals to keep up to date with the current evidence base, but an issue raised by developers is how to find this evidence base. One developer asserted that ‘*what we*'*re really keen to see is the evidence base for that impact of the built environment on health* (…) *because then we can build them in to our plans*. Another admitted knowledge translation into user-friendly guidance to be a problem: *That*'*s always going to be any research on the environment trying to get the information to the right people at the right time is always tricky* (…)’. Organisations seen by developers as able to translate the evidence base included BREAAM, Well Standard, BREAAM communities, UK Green Building Council, Building Technologies Office, Institute of Civil Engineers, Building Technologies Office (BTO).

As for the role of policy and regulations, developers doubt whether health outcomes are reflected in policy. ‘*Well I think evidence might be lacking*, *everybody builds complying with statutory obligations as a baseline*, *building regulations* etc., *but I*'*m not sure how much health is considered within building regulations*. Another also fully admits focusing on building performance:

“*A lot of properties we*'*ve just built this year are passive house*… *there are two drivers for it*. *One is the environmental sustainability*, *the zero*-*carbon issue*. *And the other one is anti*-*poverty*. *So*, *in a sense*, *these have not been influenced by a view on health*”.

While health considerations do not typically inform development practices, issues like ventilation are raised by the residents:

“*As building regulations have changed*, *the emphasis has been on energy efficiency*… *to achieve that*, *houses have had to be more sealed than they have been in the past*, *so there*'*s a lot of air tightness tests now done*. *However*, *what we probably have seen is issues with problems with condensation that can cause*, *and you can get mould*. *I do see it coming through from customers*, *you know*, *complaints about mould and damp*”.

### 4.4 Identifying the evidence base in the English planning and building policies

Five key governance tools regulating building conditions and design were identified from HHSRS (Department for Communities and Local Government (DCLG), 2006a, Department for Communities and Local Government (DCLG), 2006b), the policy review and developers' interviews:

1. National Planning Policy Framework (NPPF) and Guidance (NPPG)
2. Three Building regulations:
  a) Site preparation and resistance to contaminates and moisture: Approved Document C;
  b) Ventilation: Approved Document F. Building Regulations;
  c) Conservation of fuel and power: Approved Document LMinistry of Housing Communities and Local Government (MHCLG), 2013b; Ministry of Housing Communities and Local Government (MHCLG), 2015
3. Two British Standards Institution (BSI) Codes of Practice:

a) Control of condensation in buildings: 5250 British Standards Institute, 2009

b) protection of structures against water from the ground: 8102. British Standards Institute- BSI, 2011

4. Four Chartered Institution of Building Services Engineers (CIBSE) Guides:

a) GVA/15 Guide A: Environmental design

b) GVB1/16 Guide B1: Heating Chartered Institution of Building Services Engineers - CIBSE, 2016b

c) GVB2/16 Guide B2: Ventilation and ductwork Chartered Institution of Building Services Engineers - CIBSE, 2016a

d) GVB3/16 Guide B3: Air Conditioning and Refrigeration. Chartered Institution of Building Services Engineers - CIBSE, 2016c

5. Two EU directives.
  a) 2010/31/EU; Energy Performance of Buildings
  b) 2012/27/EU; Energy Efficiency.

In addition, wider guidance and voluntary standards (e.g. Build for Life standard, Design Council, 2015) support good practice in the field without putting pressure on developers. The figures below identified the evidence referenced in policies regulating the three HHSRS housing hazards ‘damp and mould’ (Fig. 1), ‘excess cold’ (Fig. 2) and ‘excess heat’ (Fig. 3). Of the five governance tools only two types referenced health evidence. Specifically; Building Regulation (“F: Ventilation of buildings”, MHCLG, 2013a) which referenced evidence including from the Department of Health and WHO. CIBSE (2015) Guide “A: Environmental design” also had extensive health references including WHO, DEFRA, NHS and the DoH (see Appendix A). This ranged from the most recent evidence being published in 2014 to the oldest cited evidence being from 1980.

In all of the other policies and regulations there was no related health evidence referenced. Some of the regulations referenced the Health and Safety Executive (HSE) regulations and Her Majesty's Stationery Office (HMSO) acts and regulations.

### 4.5 Identifying policy drivers of key regulations around buildings

The health, building and policy literature reviewed has identified climate change mitigation as a key driver of the building policy agenda in recent years and this provides a possible explanation as to why so little health evidence was found in statutory and key guidance documents on new residential buildings. The requirement for higher thermal specifications in buildings has resulted from obligations to mitigate climate change by reducing energy use and carbon emissions, as well as reduce fuel poverty and improve the thermal quality of homes. As shown, this has led to a conflict with some health outcomes when a balance has not been struck between energy conservation and ensuring human health.

The literature also identifies an emerging issue for the UK context that should have higher significance in building policy, planning and practice: the problem of rising temperature and its impact on current building practices. While still difficult to evaluate the actual temperature increase across the globe, human activities are estimated to have caused approximately 1.0 °C of global warming above pre-industrial levels and likely to reach 1.5 °C before 2030 (IPCC, 2018). Building regulations have made clear the need to mitigate climate change. However, research has emphasised that the predicted temperature rise in temperate and cooler countries such as the UK also requires adaptation (Mulville and Stravoravdis, 2016). Overheating in residential buildings is now identified even in the UK (Baborska-Naroznya and Grudzinska, 2016; D'Ippoliti et al., 2010; Mavrogianni et al., 2010). The Upstream project's evidence review however did not identify strong health evidence related to overheating in building (Ige et al., 2018). For health and wellbeing to become a driver of holistic policy more research is needed into the health impacts of overheating in the UK.

Historically, building policy drivers included tackling equity in health in relation to cold homes, mitigating climate change and ensuring value for money. Building regulations agencies first aimed to regulate urban development, lack of sanitation and hazards and protect the health and safety of residents (Meacham, 2016). With rising awareness of the impact of increasing greenhouse gas emissions and the significant contribution of building energy use to UK greenhouse gas emissions, the debate shifted away from protecting the residents towards protecting the planet (Mulville and Stravoravdis, 2016). Building regulations started to focus on energy conservation and performance in an effort to tackle climate change, enhance resilience and sustainability (Meacham, 2016). The result has been an increase in energy performance targets required by the UK Climate Change Act (HM Government, 2008) and the EU Energy Performance of Buildings Directive, which is implemented in the new residential buildings through building regulations (UK Part L revised in 2010 and 2013).

This shift also resulted in a positive impact on health. In particular cold homes and fuel poverty have been identified as public health issues requiring policy interventions (Poortinga et al., 2017; Camprubí et al., 2016). Energy performance in buildings became a useful tool for delivering health and equity by reducing exposure to cold (Hamilton et al., 2015). Regulatory standards to tackle climate change also informed the now defunct UK Code for Sustainable Homes (CSH) from 2007, a compulsory (CSH level 3) standard for any publicly funded building projects also used by private developers.

## 5 Discussion

This article explored the use of evidence in policy to regulate new buildings in England to deliver public health, improve climate resilience and reduce carbon footprint, in particular we explored the policy drivers and awareness of the public health evidence.

The key findings of this work are as follows:

• Review of English building policies and regulations revealed gaps in evidence use
• Building policy in England focuses on climate change mitigation rather than public health
• Building policy uses public health evidence in a patchy, unstructured way
• Lack of systems thinking has led to building standards ignoring health
• A single policy regime must regulate different phases and scales of urban development

These key findings and other issues identified in the way public health evidence is used in building policy, are discussed in more detail, below.

### 5.1 Buildings are complex and a systems approach is needed for research

Building regulations have made progress towards addressing UK climate change mitigation and fuel poverty targets. However, with global temperatures predicted to rise over the next decades, housing providers need to build for new climate circumstances and place more effort on climate adaptation. This means that policy needs to continue to regulate for improved building fabrics and technologies to save energy while addressing the unintended consequences of more insulated and air-tight buildings, that are likely to be exacerbated by climate change. Meacham (2016) has suggested developing a better understanding of holistic building performance, along with the data and tools to assess performance, and more integrated regulatory and market measures to achieve societal expectations for safe, healthy, sustainable and resilient buildings. A systems approach might help the consideration of multi-risk factors on multiple aspects of health (mental, physical, environmental, equity).

### 5.2 At the policy level, a systems approach translates into the need for policy integration and coordination

Planning and building regulations offer a range of processes applied at different stages of development and scales of the built environment. Planning policy aims at shaping the urban form, local energy production and distribution, as well as increased urban density. Meanwhile, building regulations aim at building performance (McLeod and Swainson, 2017) but are not a condition of planning enforcement.

Research demonstrates that various scales and aspects of the built environment can affect health, including those regulated by planning (e.g. neighbourhood design, appearance of buildings and landscaping and highway access and wider transport infrastructure) and those regulated by building regulations (e.g. building design to ensure safety and health, energy conservation, disabled access). Yet planning and building regulations approval regimes differ to the extent that professional bodies themselves acknowledge that clarity is lacking on what building work each regime applies to (Planning Portal/MHCLG, 2019b).

Planning policy in England has recently championed the need to create healthy communities while tackling climate change. The NPPF (MGCLG, 2018b) and NPPG (DCLG, 2012) refer extensively to the creation of healthy and sustainable communities, in particular the NPPF introducing a “*presumption in favour of sustainable development*” (MHCLG, 2018b, para. 10–11). The NPPF links planning with climate change (“*Plans should take a proactive approach to mitigating and adapting to climate change*” para. 148; MHCLG, 2018b) and also highlights the importance of quality design (“*The creation of high*-*quality buildings and places is fundamental to what the planning and development process should achieve*”, section 12, MHCLG, 2018b). Yet the NPPF does not reference public health evidence base directly.

Building regulations, which apply to new and retrofitted buildings since the 1984 Building Act (building standards are not applied retrospectively to the existing stock), as shown, make limited references to health impacts, with the exception of Part F Ventilation, which refers to the impact of mould growth and pollutants on the health of people in buildings (MHCLG, 2013a). No approved tool focused on healthy design and, as this paper has demonstrated to an integrated systems approach.

Policy priority for both NPPF and building regulations has been placed on sustainable or energy efficient design of buildings and places without sufficient consideration of health impacts. As this article has documented, this has led to unintended health consequences and is likely inadequate for building a housing stock resilient to future climate change. To address this, planning could have a wider scope to regulate specific hazards (cold, heat, damp and mould), placing more pressure on developers to take a holistic approach to energy efficiency and climate change measures that have both positive and negative impacts on the thermal quality of housing and health. A focus in both the NPPF and building regulations on the full range of SDGs that link to our urban environment could aid this holistic approach, as indeed the now defunct Code for Sustainable Homes made steps towards creating.

### 5.3 English housing market is “broken” and needs to be fixed

The market context in which these governance tools should be implemented is problematic for a number of reasons. Firstly, local authorities are under pressure to maintain their housing delivery schedule. In case of under-delivery, The NPPF 2018 states that “*the* (*local*) *authority should prepare an action plan in line with national planning guidance*, *to assess the causes of under*-*delivery and identify actions to increase delivery in future years*” (para 75, MHCLG, 2018b). This reduces their ability to negotiate on building design as well as their capacity to incorporate recent research and learning on the health impacts of different features (Carmichael et al., 2019).

Secondly, the developers are understandably unwilling to build to higher design standard for health than set out in the UK Building Regulations. Setting out higher design standards for health in the UK Building Regulations would help create “a level playing field for the private sector”, a point made in the stakeholder interviews. In particular, private sector developers (who deliver the majority of new homes in England) are answerable to their shareholders and so viability in terms of a minimum return on their investment is a key driver. While the revised 2018 NPPF puts the emphasis on assessing viability at the strategic planning level rather than project by project, the ‘need to make a profit’ barrier still exists for private developers. Therefore, additional market enablers are likely needed to make developer build to a higher design than the minimum standard.

### 5.4 The gap between expert knowledge and lay knowledge widens

Another issue is the growing gap between expert knowledge on design of energy efficient, green buildings and lay knowledge of house builders and house holders, particularly around indoor quality, and damp and mould. As design and construction of housing becomes more sophisticated with energy efficiency and carbon emission reduction key priorities, knowledge gaps widen between different groups. Firstly, the knowledge gap between building energy researcher, engineers and designers, and house builders that has contributed to a ‘performance gap’ between designed and built performance. Secondly, the knowledge gap between those who design and build homes and those who live in them, that can mean householders do not know how to effectively use more energy efficient homes. This was an issue highlighted in interviews with one stakeholder identifying the lack of knowledge about the need to open windows in more energy efficient, insulated, air-tight homes to stop mould growth.

The solutions to the first knowledge gap are complex but in terms of health impact a more holistic approach to both policy and skills training could lead to more considerate building practices where there is greater knowledge of the links between building design and health. For the second knowledge gap, improved access to education materials on new homes and how to use them could help. Even small and cheap appliances typically come with user guides, yet, the most expensive purchase in most people's lives, a home, does not. Is it time to consider mandating user guides for homes?

### 5.5 Gaps in public health evidence remain

The evidence review used in this article (Ige et al., 2018) found very limited evidence on the impact of mould, damp, cold or heat on mental wellbeing. In addition, it did not identify strong health evidence related to overheating in buildings. In the UK, no policy indicates indoor temperature levels for homes in summer that could be detrimental to health. It is difficult to conclude whether the health risks associated with living in an overheated house will be minimal or if, as an emerging concern for the UK, insufficient research has yet been carried out.

However, overheating was mentioned as a new trend in interviews and qualitative evidence review and there is a body of building energy research into the causes of overheating in homes and the possible implications of future temperature increases due to climate change (see, for example, Beizaee et al., 2013 and Gupta and Gregg, 2013). An often mentioned positive outcome of increasing temperatures is the reduction in health problems due to cold in winter. However, there are likely to still be issues with cold homes in the existing building stock, particularly in the private rented sector where improvements are dependent on private landlords. Therefore, policies and standards need to help deliver homes that are warmer in winter and cooler in summer. A focus on the health impacts of both in building policy and regulations could contribute towards a holistic approach. However, more research is needed on the wider health impacts of overheating in homes, particularly in the UK context.

With the exact extent of future climate change and the impact on the number of too cold and overheated homes in the UK uncertain, preparing for a warmer climate in England is a moving target for developers, without policy guidance. Yet, for purely commercial reasons developers cannot afford to ignore climate change and its impact on housing due to the future impact on house prices and their reputation. Developers will be eager to know the impact of overheating and respond to new demands on the market.

## 6 Conclusion

A key challenge identified has been the lack of systems approach and integrated policy environment to take into account all the factors and actors influencing the building policy process. Interviews from developers confirm that finding user-friendly evidence means relying on expert bodies. Building regulations can provide clarity for builders, and are not dependent on the competency of local authorities to interpret and integrate health evidence into building design. Yet health evidence needs to filter through the policy process and the research has identified a stark lack of influence of health evidence.

Research findings in this article further support the need for a holistic revision on the use of evidence in policy and regulation that considers interdependencies, and includes a specific focus on public health, rather than simply on environmental quality, climate resilience or reducing the carbon footprint of buildings in isolation.

In the future, to create a more holistic policy approach to housing – design, build and planning – an integrated framework is needed with a broader set of key drivers. A framework based on the sustainable development goals (SDGs) where indicators link to the built environment (as set out in the introductory section) could help ensure sufficient importance is placed on energy efficiency and climate change, equity, sanitation, cost effectiveness, and health and wellbeing. The aim would be to ensure firstly that unintended health consequences of focussing on one driver, such as energy efficiency (discussed in this article), are avoided and secondly to improve the overall health and wellbeing impact of homes.

In view of the strong evidence base linking housing design and health, the next step of the research is to further review the scope and limit of public health evidence to inform policy and regulation in relation to a wider range of housing hazards. Based on findings in this case study over the thermal quality of homes, greater consideration of the public health evidence base is needed in (re)development of building regulations and other standards. Ideally, this should happen at the national level with a holistic review of the buildings regulations. At the local level, further work is needed to better contextualise the health evidence for local authorities in relation to local planning, and house building where local authorities are in the developer role.

By highlighting the available evidence and policies related to health and built environment, the article contributes towards closing the gap between SDGs and the relevant data. Furthermore, SDGs serve as a constant reminder that monitoring and evidencing key aspects of urban living will facilitate effective interventions. By compiling indicators related to both planetary and human health, SDGs emphasise the need for a systemic account of building regulations and practices. Finally, as the SDGs Agenda aims to enable sustainable development by 2030, the paper highlights the urgency for action and the requirement to consolidate the existing evidence and translate it to the practitioner-friendly formats, that could use the SDGs as a framework.

The following is the supplementary data related to this article.

Appendix A Public health evidence referenced in the five key policy instruments explored in Fig. 1, Fig. 2, Fig. 3. Appendix A

## Funding

This work was supported by the Wellcome Trust through the Wellcome Trust Sustaining Health Award (Award number: 106857/Z/15/Z).

## Declaration of competing interest

None.

## Footnotes

1 BRE defines “poor housing” after extending the definition to include 3.5 million (15%) of the poorest housing stock in the country identified with a significant HHSRS hazard (BRE, 2015).

2 The five environment themes included: buildings, transport, neighbourhood design, food and natural environment.
