# Supplementary material for: Healthy buildings for a healthy city: Is the public health evidence base informing current building policies?
Source: Sci Total Environ. 2020 Jun 1;719:137146. doi: 10.1016/j.scitotenv.2020.137146 (PMC7166076; doi:10.1016/j.scitotenv.2020.137146)
Supplement: Appendix A — Public health evidence referenced in the five key policy instruments explored in Fig. 1, Fig. 2, Fig. 3. [file mmc1.docx]

**Appendix A: Public health evidence referenced in the five key policy instruments explored in figures 1-3.**

Arundel A.V., Sterling E.M., Biggin J.H. and Sterling T.D. (1986) ‘Indirect health effects of relative humidity in indoor environments’ *Environmental Health Perspectives,* 65, pp. 351–361.

Berry B. and Flindell I. (2009) *Estimating Dose-Response Relationships Between Noise Exposure and Human Health in the UK* (London: Department for Environment, Food and Rural Affairs) (available at http://archive.defra. gov.uk/environment/quality/noise/igcb/publications/healthreport.htm) Accessed: 25^th^ September 2019

Department of Health (DoH) (2012) *Vitamin D: Advice on Supplements for At Risk Groups*, Letter of Chief Medical Officers for the UK to Health Professionals [online] (London: Department of Health) (available at [www.gov.uk/government/publications/vitamin-dadvice-on-supplements-for-at-risk-groups](http://www.gov.uk/government/publications/vitamin-dadvice-on-supplements-for-at-risk-groups)) Accessed: 25^th^ September 2019

Department of Health Committee on the Medical Effects of Air Pollutants (1998) *Quantification of the Effects of Air Pollution on Health in the United Kingdom* (London: TSO)

Department of Food and Rural Affairs (DEFRA) (2011) *The Air Quality Strategy for England, Scotland and Northern Ireland* (London: DEFRA) Available at: https://www.gov.uk/government/ publications/the-air-quality-strategy-for-england-scotland-wales-andnorthern-ireland-volume-1 Accessed: 25^th^ September 2019

European Environment Agency (EEA) (2010) *Good practice guide on noise exposure and potential health effects*. Technical Report No. 11/2010 (Copenhagen: European Environment Agency) (available at <http://www.eea.europa.eu/publications/goodpractice-guide-on-noise>) Accessed: 25^th^ September 2019

Fisk W.J. (2000) ‘Health and productivity gains from better indoor environments and their relationship with building energy efficiency’ *Annual Review of Energy and the Environment,* 25 (1), pp. 537–566

Fisk W.J., Lei-Gomez Q. and Mendell M.J. (2007) ‘Meta-analyses of the associations of respiratory health effects with dampness and mold in homes’ *Indoor Air*. 17, pp. 284–296

Goodman T.M., Gibbs D.R. and Cook G. (2006) Report DQL-OR 019: *Better Lighting for Improved Human Performance, Health and Well-being and Increased Energy Efficiency*: A Scoping Study for CIE-UK (Teddington: NPL)

Haghighat F., Megri A.C., Donnini G. and Giorgi G. (2000) ‘Responses of disabled, temporarily ill, and elderly persons to thermal environments’ ASHRAE Trans. 106 (2), p. 329

Ising H. and Kruppa B. (2004) ‘Health effects caused by noise: Evidence in the literature from the past 25 years’ *Noise and Health.* 6 (22), pp. 5–13.

Jaakkola M.S., Haverinen-Shaughnessy U., Douwes J. and Nevalainen A. (2011) ‘Indoor dampness and mould problems in homes and asthma onset in children’ in Braubach M, Jacobs D and Ormandy D (eds.) *Environmental Burden of Disease Associated with Inadequate Housing: Methods for Quantifying Health Impacts of Selected Housing Risks in the WHO European Region* (Copenhagen: WHO Regional Office for Europe)

Jones K. (2010) *Environmental Noise and Health*: A Review ERCD Report 0907 (London: Civil Aviation Authority)

Kales S.N., Islam T. and Kim M. (2011) ‘Household carbon monoxide poisoning’ in Braubach M, Jacobs D and Ormandy D (eds.) *Environmental Burden of Disease Associated with Inadequate Housing: Methods for Quantifying Health Impacts of Selected Housing Risks in the WHO European Region* (Copenhagen: WHO Regional Office for Europe)

Mudarri D. and Fisk W.J. (2007) ‘Public health and economic impact of dampness and mold’ *Indoor Air Journal*. 17, pp. 226–235

NHS (2009) *Saving Carbon, Improving Health* (Cambridge: National Health Service Sustainable Development Unit)

Parsons K.C. (2003) *Human Thermal Environments: the effects of hot, moderate, and cold environments on human health, comfort, and performance* (2nd edn.) (London: Taylor & Francis)

Sieber W.K., Petersen M.R., Staynor L.T., Malkin R., Mendell M.J., Wallingford K.M., Wilcox T.G., Crandall M.S. and Reed L. (1996) ‘Associations between environmental factors and health conditions’ *Proc. Conf. Indoor Air 2*, pp. 901– 906

Stansfield S. and Matheson M. (2003) ‘Noise Pollution: Non-Auditory Effects on Health’ *British Medical Bulletin.* 68 (1), pp. 243–257

Wilkinson, P., Smith, K.R., Beevers, S., Tonne, C. & Oreszczyn, T. (2007) Energy and Health 4: Energy, energy efficiency, and the built environment, *The Lancet,* 370 (9593), pp. 1175.

WHO (1980) Environmental Health Criteria, Section 12: Noise (WHO: Geneva)

WHO (1982) Indoor air pollutants: exposure and health effects Report of WHO meeting, Norlinger, 8–11 June 1982 (Copenhagen: World Health Organization Regional Office for Europe) (available at <http://whqlibdoc.who.int/euro/r&s/EURO_R&S_78.pdf>) Accessed: 25^th^ September 2019

WHO (1987) Health impact of low indoor temperatures (report on a WHO meeting) (Copenhagen: WHO)

WHO (1999) Guidelines for community noise (Geneva: WHO)

WHO (2000) Air Quality Guidelines for Europe European Series 91 (Copenhagen: WHO)

WHO (2011) Burden of disease from environmental noise: Quantification of healthy life years lost in Europe (Copenhagen: World Health Organization Regional Office for Europe) (available at <http://www.euro.who.int/__data/assets/pdf_file/0008/136466/e94888.pdf>) Accessed: 25^th^ September 2019
